# Influence of Mesenchymal Stem Cell Transplantation on Stereotypic Behavior and Dopamine Levels in Rats with Tourette Syndrome

**DOI:** 10.1371/journal.pone.0062198

**Published:** 2013-04-26

**Authors:** Xiumei Liu, Xueming Wang, Lixia Li, Haiyan Wang, Xiaoling Jiao

**Affiliations:** Department of Pediatrics, Yuhuangding Hospital of Qingdao University, Yantai, Shandong, China; University of Medicine and Dentistry of New Jersey, United States of America

## Abstract

**Context:**

Tourette syndrome (TS) is a heterogeneous neuropsychiatric disorder. Chronic motor and phonic tics are central symptoms in TS patients. For some patients, tics are intractable to any traditional treatment and cause lifelong impairment and life-threatening symptoms. New therapies should be developed to address symptoms and overt manifestations of TS. Transplantation of neurogenic stem cells might be a viable approach in TS treatment.

**Objective:**

We used mesenchymal stem cell (MSC) transplantation to treat TS. We discuss the mechanism of action, as well as the efficiency of this approach, in treating TS.

**Settings and Design:**

An autoimmune TS animal model was adopted in the present study. Forty-eight Wistar rats were randomly allocated to the control group and the 2 experimental groups, namely, TS rats+vehicle and TS rats+MSC. MSCs were co-cultured with 5-bromodeoxyuridine (BrdU) for 24 h for labeling prior to grafting.

**Methods:**

Stereotypic behaviors were recorded at 1, 7, 14, and 28 days after transplantation. Dopamine (DA) content in the striatum of rats in the 3 groups was measured using a high-performance liquid chromatography column equipped with an electrochemical detector (HPLC-ECD) on day 28 after transplantation.

**Statistical analysis:**

Statistical analysis was performed by repeated measurements analysis of variance to evaluate stereotypic behavior counts at different time points.

**Results:**

TS rats exhibited higher stereotypic behavioral counts compared with the control group. One week after transplantation, TS rats with MSC grafts exhibited significantly decreased stereotypic behavior. Rats with MSC grafts also showed reduced levels of DA in the striatum when compared with TS rats, which were exposed only to the vehicle.

**Conclusions:**

Intrastriatal transplantation of MSCs can provide relief from the stereotypic behavior of TS. Our results indicate that this approach may have potential for developing therapies against TS. The mechanism(s) of the observed effect may be related to the suppression of DA system by decreasing the content of DA in TS rats.

## Introduction

Tourette syndrome (TS) is a developmentally regulated neurobehavioral disorder in which chronic motor and phonic tics are central symptoms. The prevalence of TS is estimated to be between 4 and 6 per 1,000 children and adolescents [1]. Epidemiological studies have shown that TS is relatively common and more prevalent in boys. Studies show that approximately 50% of TS patients continue to suffer from tics well into adulthood, and at least a third of patients with TS exhibit tic-related self-injurious behavior [2]. For some individuals, tics can cause lifelong impairment, and about 5% of TS patients have life-threatening symptoms, which are defined as malignant TS [3].

The pathophysiology and etiology of TS are unclear; however, it is possible that a combination of genetic and environmental factors is involved in it [4]. Structural and functional neuroimaging and neurophysiological and post-mortem studies have shown that the basal ganglia and related cortico-striato-thalamo-cortical circuits, as well as the dopaminergic neuronal system, may be dysfunctional in TS [5], [6]. Traditional therapies such as pharmacological treatments, behavioral therapies, and surgical approaches can reduce the frequency and intensity of tics but cannot eliminate them entirely. Therefore, it is recommended that treatments suggested above should be regarded only as symptomatic therapy. Surgical techniques involving deep brain stimulation (DBS) of the thalamus or globus pallidus may also be considered for patients with severe TS. However, side effects of DBS, such as drowsiness, reduced energy, psychosis, and spontaneous tic recurrence, have been reported [7], [8]. Furthermore, the use of DBS for the treatment of TS is limited because of post-surgical complications and the necessity of expensive infrastructure to provide this form of therapy [9]. Therefore, new therapeutic options should be explored for TS patients who are significantly impaired by this syndrome.

In recent years, stem cell-based therapy has been perceived as a potential treatment for many neurological disorders. Experiments with animal models suggest that if stem cells are injected into the brain or even the bloodstream, the transplanted cells can survive and migrate to damaged portions of the nervous system, following which they get incorporated into working neural circuits and replace dead neurons. This ability is extremely important for the reconstitutive effects of stem cell treatments. The tested animals display significant improvement in various functions, resulting from stem cell transplants [10]. Stem cell therapy may provide a breakthrough for some of the existing limitations of traditional pharmaceutical approaches, and it is a good choice for the treatment of neural diseases whose exact pathogenesis is unclear [11]. Neural stem cells (NSCs) are considered to be a heterogeneous population of mitotically active, self-renewing, multipotent, and immature progenitor cells [10]. In 2008, we transplanted NSCs into the striatum of TS rats and observed the therapeutic effects of NSCs on the stereotypic behaviors of TS rats. NSCs survived in the brain of TS rats, and a fraction of them differentiated into neurons and gliocytes [12]. Compared with NSCs, mesenchymal stem cells (MSCs) are a better choice for cell transplantation therapy because they are easily accessible, capable of rapid expansion in culture, immunologically inert, and capable of long-term survival and integration with the host tissue. In this study, we propose transplantation of MSCs as a novel therapy for TS and provide insights into the mechanism of action of these cells.

Although the definitive pathophysiological mechanism of TS is not well understood, it is widely believed that abnormalities in the dopaminergic neuronal system play a primary role in the pathophysiology of TS [13]. The efficacy of anti-dopaminergic agents such as haloperidol in treating TS, along with other clinical and basic science findings, have contributed to the concept that abnormal dopamine (DA) signaling and aberrations in basal ganglia processing are important factors contributing to the pathophysiology of TS [14]. In the present study, we transplanted MSCs into the striata of TS rats and subsequently investigated the effect of the transplanted MSCs on stereotypic behaviors and DA levels of TS rats.

## Subjects and Methods

### Animals

Forty-eight 7–8-week-old Wistar rats (24 of each sex; weight range, 205–220 g) were used for this study. Animals were housed in an environmentally controlled room maintained at a temperature of 21°C ±1°C with a 12-h light/dark cycle (lights on 0700–1900 hours). The animals had free access to food and water. Experimental procedures were performed in accordance with the NIH Guidelines for the care and use of laboratory animals.

### MSC Preparation and Flow Cytometric Analysis

Mononuclear cells were isolated from the long bones of 5 adult Wistar rats. The bones were dissected free, and the proximal and distal ends were removed to reveal the marrow cavity. Cells were cultured in low-glucose Dulbecco’s-modified Eagle’s medium (Gibco-BRL, Grand Island, NY, USA) supplemented with 15% fetal bovine serum (Gibco-BRL), 100 U/mL penicillin, and 100 µg/mL streptomycin (Gibco-BRL). Cells were incubated in 5% CO_2_ at 37°C. Culture medium was replaced every 3–4 days. When the cells reached confluence, adherent cells were harvested with trypsin (Sigma, St. Louis, MO, USA) and subsequently passaged. A small part of the MSC samples from the third to fourth passages was tested utilizing flow cytometric analysis. The remaining MSCs were co-incubated with BrdU for 48 h prior to transplantation. To label MSCs, bromodeoxyuridine (BrdU, 10 µg/mL, Sigma, St. Louis, MO, USA)), a thymidine analog and marker of newly synthesized DNA, was added into the medium for 48 h before transplantation. More than 90% of MSCs selected for immunostaining were immunoreactive for BrdU. Subsequently, cells were harvested and resuspended in PBS at a density of 1×10^5^ cells/µL and stored on ice until grafting.

Cells in their third to fourth passage were collected and treated with 0.25% trypsinase. Cells were stained with fluorescein isothiocyanate- or phycoerythrin-conjugated anti-marker monoclonal antibodies in 100 µL PBS for 30 min at 4°C, as suggested by the manufacturer. Antibodies against rat antigens CD29, CD34, CD44, and CD45 were purchased from SeroTec (Raleigh, NC, USA). Appropriate isotype-matched, non-reactive, fluorochrome-conjugated antibodies were used as controls. Cells were analyzed using a flow cytometry system (Cytometer 1.0, CytomicsTM FC500, Beckman Coulter, Los Angeles, CA, USA).

### Animal Preparation and in vivo Surgery

A total of 48 Wistar rats were randomly divided into 3 groups: control group, TS+vehicle (PBS) group, and TS+MSC group (n = 16 for each group). Surgery was performed as previously described [15], [16]. Briefly, rats were deeply anesthetized with chloral hydrate (400 mg/kg, i.p.) and placed in a stereotaxic apparatus (Stoelting, Wood Dale, Illinois, USA) with the incisor bar set at 3.5 mm below the interaural line. Using aseptic surgical techniques, the skull was exposed, holes were drilled where appropriate, and 28-gauge guide cannulae were implanted into the bilateral striata [15]. Coordinates for cannula placements were 2.0 mm anterior-posterior from the bregma and 4.0 mm medial-lateral and –7.0 mm dorsoventral from the skull. Proper post-surgical care was provided to the animals. The diet of operated rats was supplemented with fresh fruit and egg yolk to maintain body weight.

Rats were allowed to recover for 1 week to re-establish integrity of the blood brain barrier. After the recovery period, osmotic mini-pumps (Alzet, Palo Alto, CA, USA) filled with PBS were connected to each cannula using a polyethylene tube loaded with 50 µL undiluted sera of TS patients using sterile conditions. All sera of TS patients (twelve male subjects and four female subjects, age range = 8–14 years, mean = 11.3) were taken from our serum bank. No subjects were taking psychostimulants at the time that blood was drawn. Collection of sera was performed under a protocol approved by the Institutional Review Board and after consent was obtained. The sera of TS patients bear a high titer of antibasal ganglia antibody, which could induce impairments of the striatum and subsequent stereotypic behavior. The antibasal ganglia antibody ELISA optical density readings of 16 TS subjects selected for microinfusion was 0.952±0.184. Sera were microinfused at a rate of 0.5 µL/h for 72 h. Control surgery rats were microinfused with PBS. After 72 h, animals were again sedated with an intraperitoneal injection of chloral hydrate (100 mg/kg) and placed in the stereotaxic frame. An incision was made along the midline to expose the skull, and the pumps were removed. The MSC suspension (10^5^/µL, 5 µL/site) was bilaterally injected into the serum-infusion site. The needle was maintained in place for 5 min before slow removal. The wound was closed with a surgical suture. Each grafted animal received a total of 10^6^ MSC. For control grafting, animals underwent the same grafting procedure but received a vehicle infusion of PBS of equal volume. Animals were intramuscularly administered 65,000 units of sodium penicillin and were maintained on a thermal pad until they awoke. The rats were then returned to the home cages.

### Assessment of Stereotypic Manifestation of TS

In this study, we established an autoimmune animal model of TS according to protocols established by Hallett and Taylor [15], [16]. Stereotypic behaviors in rats were similar to motor and phonic tics in TS patients, and therefore, stereotypy was used as an indicator for successful induction of TS in these animals. Rat movements were video- and audio-taped at the end of the light cycle for 30 min. Stereotypy was recorded, including biting (teeth touching cage or wood chips, vacuous chewing on other objects except the body), taffy pulling (forepaw to the mouth and face), self-gnawing, licking, grooming, head shaking, paw shaking, rearing, and episodic utterances [15], [16]. Grooming behavior was recorded by the number of minutes in which grooming occurred. Episodic utterance was defined as repeated medium-pitched sounds of short duration. Stereotypic movements were recorded at 1, 7, 14, and 28 days after transplantation. Animals received a total score equal to the sum of observed movements. A researcher, trained to identify stereotypy of interest and blind to the types of grafts and substance microinfused, quantified the stereotypy by reviewing and listening to the video tapes.

### Immunohistochemistry

Twenty eight days after MSC transplantation, rat brains were removed from the cranium and post-fixed in paraformaldehyde prior to sectioning [7]. Rat brain sections were embedded in paraffin and 6-µm thick coronal sections were prepared. All immunostaining processes were performed according to instructions from the HistostainTM-DS kit (Zhongshan, Peking, China). Sections were incubated in 3% H2O2 in methanol for 10 minutes and in 10% goat serum for 10 minutes at room temperature. The sections were incubated in rabbit anti-rat BrdU monoclonal antibody (Zhongshan, Peking, China; 1∶250) at 4°C overnight in a humidity chamber and were then treated with biotinylated goat anti-rabbit IgG (Zhongshan; 1∶200) for 10 minutes. The remaining primary antibodies, which included mouse anti-rat GFAP monoclonal antibody (Sigma, USA; 1∶200, astrocyte marker), mouse anti-rat MAP2 monoclonal antibody (Sigma, USA; 1∶200, neuronal marker), and mouse anti-rat Nestin monoclonal antibody (BD Bioscience Pharmingen, Newark, NJ, USA; 1∶400, neural precursor cell marker) were added. Subsequently, the sections were treated with biotinylated secondary antibody goat anti-mouse monoclonal IgG (1∶100). Finally, horseradish peroxidase-conjugated streptavidin was added and sections were coverslipped in permanent mounting medium.

### High-performance Liquid Chromatography with Electrochemical Detection

Twenty-eight days after MSC transplantation, rats in the 3 groups described were killed by decapitation. Both sides of the striatum were isolated and transferred to liquid nitrogen for storage. Samples were homogenized in ice-cold perchloric acid (0.1 mol/L) with 1% ethanol and 0.02% ethylenediamine tetra-acetic acid. DA in the dialysates was measured using a high-performance liquid chromatography column equipped with an electrochemical detector. The HPLC-ECD system comprised a reverse-phase column (MA-5 ODS, 150×4.6-mm ID, Eicom), a model L-6000 pump (Hitachi), and an electrochemical detector (ECD-100, Eiercom). The mobile phase contained 85 mmol/L citric acid–100 mmol/L sodium acetate, 0.2 mmol/L disodium EDTA, 1.2 mmol/L sodium octane sulfonate, and 5% methanol in deionized and distilled water. The flow rate was 1 mL/min, and the pH was adjusted to 3.5. Results were expressed as ng/mg wet weight of brain tissue.

### Statistical Analysis

All statistical analyses were carried out using SPSS (version 13.0 for Windows; SPSS Inc, Chicago, IL, USA). Data were reported as mean ± SD. Statistical analysis was performed by repeated measurements analysis of variance to evaluate stereotypy counts at different time point. A *P* value of <0.05 was considered statistically significant.

## Results

### Assessment of Stereotypy

**Figure 1:**
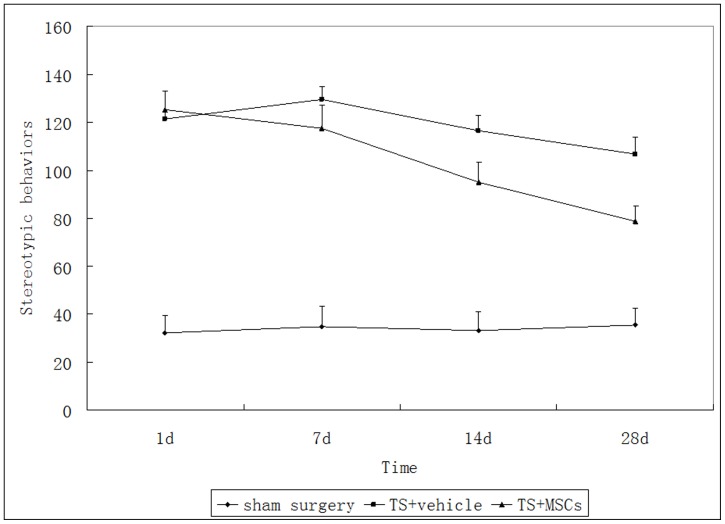
Stereotypy counts were recorded for 30 min at different time points in 3 groups. Scores are higher in the Tourette syndrome (TS) group than those in the control group. TS rats with mesenchymal stem cell (MSC) grafts exhibited decreased stereotypic behaviors compared with TS+vehicle rats. *P*<0.05.

Serologic studies of TS have demonstrated that the existence of anti- basal ganglia antibodies induced striatal dysfunction. Stereotypic behaviors were successfully induced in rats by intrastriatal microinfusion of the sera of TS patients. Rats were observed at 1, 7, 14, and 28 days after transplantation. Stereotypic behaviors were recorded and quantified during a 30-min observation period. Using repeated measurements analysis of variance, the overall model exhibited significant group (F = 43.58, P<0.01) and day effects (F = 18.42, P<0.01), as well as (group×day) interactions (F = 14.25, P<0.01), indicating varying degrees of differences among groups and across days. Statistical analysis suggested that TS rats exhibited higher stereotypic behavioral counts compared with the control group. TS+MSC and TS+vehicle injection groups exhibited significant differences between the groups (F = 35.72, P<0.01), as well as a time effect (F = 40.36, P<0.01). TS rats with MSC grafts exhibited significantly decreased stereotypic behaviors 1 week after transplantation. (Figure 1).

### Characteristics of MSCs

**Figure 2:**
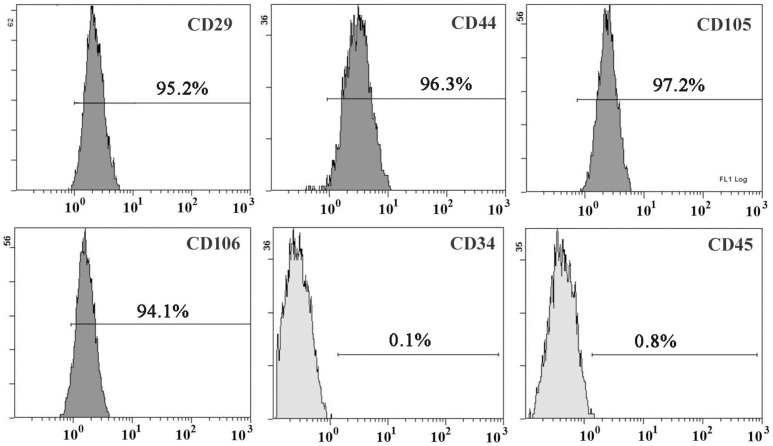
Flow cytometry analysis of MSCs. Cells were cultured for 3–4 passages, harvested, and analyzed by a flow cytometry system. The y-axis represents the number of cells, and the x-axis represents CD29, CD-44, CD105, CD106, CD34, and CD45.

Bone marrow contains a category of non-hematopoietic multipotent cells that can be cultivated *in vitro*. They were initially called plastic adherent cells but have recently been renamed as bone marrow stromal cells. In this study, primary MSC cultured as plastic adherent cells were maintained in culture. After 14 days in culture, the attached MSCs developed into an adherent layer with abundant dispersed fibroblast-like cells; each colony was formed by fibroblast-like cells. By day 28, MSCs had proliferated and formed a nearly continuous layer comprising mainly of fibroblast-like cells amongst which a subset of characteristically flattened and spindle-shaped-cells could be recognized. MSCs are mesenchymal elements that provide structural and functional support for hemopoiesis. These cells lack the hematopoietic surface markers but express mesenchymal markers. Our flow cytometry results demonstrate that cultured cells were negative for CD34 and CD45 (hematopoietic lineage markers). These cells showed strong expression of integrin CD29, endothelial progenitor/precursor lineage marker CD105, and matrix receptors CD44 and CD106 (Fig. 2).

### Immunohistochemistry

**Figure 3:**
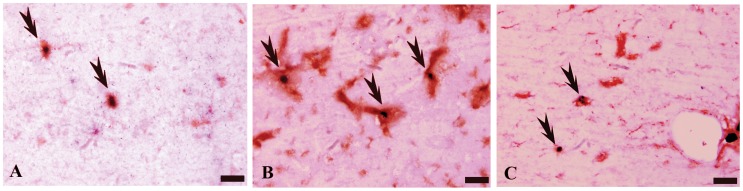
Immunostaining analyses of grafted MSCs in a rat model of Tourette’s syndrome at 4 wk after transplantation. Grafted MSCs are identified by BrdU staining. (A) Grafted cells are double-labeled with anti-BrdU (purple) and anti-Nestin (red). (B) Some grafted MSCs differentiated into neurons, as evidenced by double-labeling with BrdU and MAP-2. (C) Grafted cells differentiated into astrocytes, which coexpressed BrdU and GFAP. Scale bar: 20 µm. Arrows represent transplanted MSCs that differentiated into different types of neural cells.

In the TS rats with MSC graft, 5-bromodeoxyuridine (BrdU)-positive cells were prevalent in the striatum near the injection site. BrdU and Nestin double-positive cells were also observed, indicating that grafted cells differentiated into neural precursor cells (Figure 3A). Results also showed that a considerable portion of transplanted cells coexpressed BrdU and microtubule-associated protein 2 (MAP-2) (Figure 3B), and several coexpressed BrdU and glial fibrillary acidic protein (GFAP) (Figure 3C).

### Levels of DA in the Striatum

**Figure 4:**
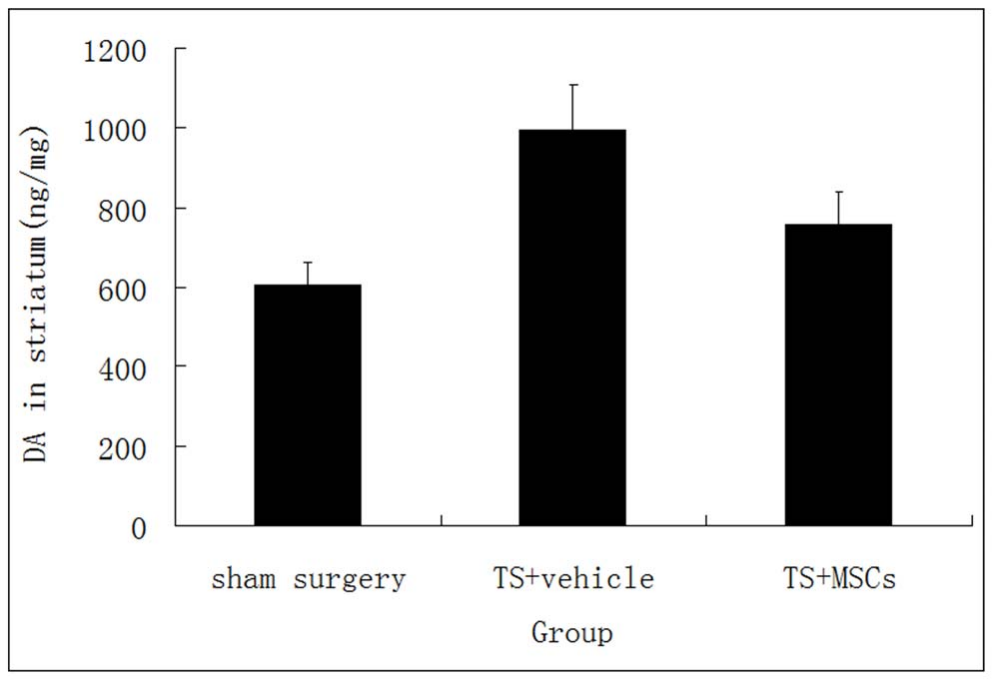
The amounts of dopamine (DA) in striatum homogenate (Figure 4) were analyzed at the end of the test by high-performance liquid chromatography with electrochemical detection in 3 groups (n = 16).

Hyperfunction of the DA system plays an important role in the etiopathogenesis of TS. The content and activity of DA in the striatum are closely associated with TS. Some studies have shown that DA levels are increased in TS patients [13]. At the end of our experiments, DA levels in the striatum of TS rats were significantly increased compared with those from the control surgery group (994.8±112.6 ng/g versus 607.3±56.6 ng/g, P<0.01). Reduction in the levels of DA or enhanced degradation of DA is likely to control the stereotypic behavior seen in TS. TS rats that received the MSC grafts had lower DA levels compared with untreated rats in the TS group (757.9±82.4 ng/g versus 994.8±112.6 ng/g, P<0.01). (Figure 4).Our findings demonstrate that transplantation of MSCs can reduce the levels of DA in TS rats and alleviate symptoms of TS.

## Discussion

The prevalence of TS is estimated to be approximately between 4 and 6 per 1,000 children and adolescents [17]. In general, tics are self-limited or can be treated by behavioral or pharmacological therapy. It is estimated that approximately 20% of children with TS continue to experience a moderate level of impairment of global functioning by the age of 20 years [18]. Furthermore, severe tics can lead to physical injury and significant functional impairment. Patients with malignant TS have life-threatening and self-injurious symptoms, which are intractable to both conservative treatments and neurosurgical procedures such DBS. In order to find effective new therapies for these unfortunate patients, we have examined the approach of transplanting MSCs into the brain of rats, which serves as an animal model for TS. Our results published earlier show that transplantation of NSCs into TS rats resulted in improvement of stereotypic behaviors in TS rats [12].

In recent years, MSCs have shown great therapeutic potential in many neurological diseases. Direct or intravascular MSC transplantation has been shown to bring about functional improvements in experimental animal models of central nervous system (CNS) injury and disease [19]. Furthermore, owing to their relatively easy isolation from bone marrow and their extensive capacity for *in vitro* expansion, transplantation of MSCs has been considered as a good choice for cell therapy and tissue re-engineering. In a previous study, we found that TS rats bearing MSC grafts exhibited significantly decreased stereotypic behaviors compared with control animals [20]. Research suggests that under specific experimental conditions, MSC can differentiate into cells with neural phenotypes, which would potentially enable them to replace neural tissue lost after CNS injury. In the present study, BrdU and Nestin double-positive cells were quantified in the rat striatum following MSC transplantation, which demonstrated that grafted MSC differentiated into neural precursor cells. Some of transplanted cells coexpressed BrdU and MAP-2 and several coexpressed BrdU and GFAP, which demonstrated that transplanted MSCs differentiate into neurons and astrocytes. The cell replacement theory was based on replacement of damaged neural cells with alternative functioning cells that induce long-lasting, clinical improvement. It is reasoned that transplanted cells survive, integrate into the endogenous neural network, and lead to functional improvement. These results led us to speculate that replacement of neuronal cells by MSCs contributed to the functional improvement of TS rats. However, the rate of differentiation of MSCs was lower than we expected it to be. This fact makes it difficult to assess evidence for the mechanism of action of MSCs in alleviating symptoms of TS.

The pathophysiology and etiology of TS are not completely understood. Brain imaging, neurophysiology, and post-mortem studies support the involvement of cortical-striatal-thalamo-cortical pathways in the progression of TS. The clinical efficacy of DA antagonists in tic suppression gives rise to speculation that abnormalities of striatal dopaminergic neurotransmission are part of the underlying mechanism that results in TS [21]. Since it is widely believed that abnormalities of DA neurotransmission play a primary and important role in the pathophysiology of TS, in this study, we examined the levels of DA in TS rats after transplantation of MSC.

DA is a major monoaminergic neurotransmitter released by nerve terminals originating from midbrain neurons. Metabolism of DA in nerve terminals is regulated by multiple processes such as synthesis, storage, reuptake by presynaptic cells, and catabolism. Since these metabolic processes are energy-dependent, DA metabolism in the striatum is disrupted when the striatal region is damaged [22]. It has been demonstrated that excessive release of DA takes place into the extracellular space in brain ischemia animal models [23]. In our study, the autoimmune TS animal model was microinfused with anti-basal ganglia antibody, which induced basal ganglia impairment. Our results show that DA levels were high in the striatum of TS rats. These results concur with those from other studies as well [22], [23].

DA modulates the activity of neurons in the striatum by binding to and signaling via DA receptors. There are 2 families of DA receptors, namely, D1-like receptor (DRD1) and D2-like receptor (DRD2). The content and activity of DA and density and sensitivity of DRD2 in the striatum seemed to be closely associated with TS. DA produced a remarkable effect only after binding to DRD2. Many studies have shown that levels of DA and activity of the DRD2 receptor are increased in TS patients [24]. It is suggested that decreasing the levels of DA or promoting its metabolism and inhibiting the activity of the DRD2 receptor can control the associated stereotypic behavior [25]. In the present study, we demonstrate that following the transplantation of MSC, the levels of DA in TS rats were decreased and the stereotypic behaviors were suspended.

The present study has several limitations. First, this study was an *in vivo* rat study, and caution should be exercised when applying these results to human patients in clinical settings. Second, this research was based on a transient and induced TS model wherein it was difficult to reproduce the full spectrum of pathogenesis of TS as seen in humans. Future studies are needed to examine the effects of MSC transplantation on the dopaminergic nervous system.

### Conclusion

In summary, the results of this study provide evidence that transplantation of MSCs effectively inhibits stereotypic TS behaviors. The mechanism of rectification of this pathogenesis is likely to be achieved by downmodulating the activity of dopaminergic neurons.
